# Culture Dimensionality Regulates Protein Expression and Bioactivity in THP-1-Derived Macrophages

**DOI:** 10.3390/biomedicines14040882

**Published:** 2026-04-13

**Authors:** Shang-Wun Jhang, Liang-Fang Lin, Gizem Naz Canko, Bill Cheng

**Affiliations:** 1 Department of Neurosurgery, Changhua Christian Hospital, Changhua 500, Taiwan; 133393@cch.org.tw; 2 Graduate Institute of Biomedical Engineering, National Chung-Hsing University, Taichung 402, Taiwan; lfanny8938@gmail.com (L.-F.L.); gizemcanko@gmail.com (G.N.C.); 3 Ph.D. Program in Tissue Engineering and Regenerative Medicine, National Chung-Hsing University, Taichung 402, Taiwan

**Keywords:** monocyte-derived macrophages, 2D & 3D cultures, protein expressions, bioactivity

## Abstract

**Background/Objectives**: Macrophage phenotype and function are highly sensitive to environmental cues; however, most in vitro studies rely on 2D culture systems that lack physiologically relevant structural context. The spatial dimensionality can influence immune cell signaling, yet the roles of these cells in regulating macrophage behavior remain incompletely understood. This study aimed to investigate how cultural dimensionality affects the phenotype, signaling, and functional activity of monocyte-derived macrophages. **Methods**: GFP-expressing THP-1 monocytes were differentiated into M0, M1, and M2 macrophages and cultured either on planar substrates or within 3D matrices composed of Matrigel or type I collagen. Macrophage morphology and viability were monitored. Membrane receptor expression and secreted cytokines were examined and quantified. Functional activity was further assessed through coculture experiments with RFP-expressing MDA-MB-231 breast cancer cells. **Results**: Compared with 2D culture, 3D environments induced distinct morphological and viability changes in macrophages. Collagen matrices supported sustained growth, subtype-specific morphologies, and enhanced functional activity, whereas Matrigel promoted aggregation and reduced viability. Core lineage markers remained stable across conditions, but activation-associated receptors and cytokine profiles were strongly influenced by dimensionality. 3D culture enhanced TNF-α expression and altered serglycin glycosylation patterns. In coculture assays, macrophage effects on tumor cell growth depended on polarization state and were more pronounced in 3D systems. **Conclusions**: These findings demonstrate that culture dimensionality and ECM composition are key regulators of macrophage phenotype and function. Collagen-based 3D systems better reproduce physiologically relevant macrophage behaviors than conventional 2D platforms, highlighting the value of structurally biomimetic models for immunological studies and therapeutic screening.

## 1. Introduction

Macrophages are highly plastic innate immune cells that play central roles in host defense, tissue homeostasis, wound repair, and tumor progression. In response to environmental cues, monocytes differentiate into macrophages that adopt distinct functional phenotypes along a polarization spectrum, commonly simplified as pro-inflammatory (M1-like) and anti-inflammatory or reparative (M2-like) states. These phenotypes differ in morphology, receptor expression, cytokine production, and effector function, enabling macrophages to dynamically adapt to tissue-specific demands [1]. Although soluble factors such as cytokines and pathogen-associated signals are well-recognized drivers of macrophage polarization, accumulating evidence indicates that physical and structural properties of the cellular microenvironment also critically influence macrophage behavior [2].

In vivo, macrophages reside within complex three-dimensional (3D) extracellular matrix (ECM) networks that provide not only biochemical ligands but also mechanical and spatial cues. These cues regulate adhesion signaling, cytoskeletal organization, migration, metabolism, and transcriptional programs [3]. Conventional in vitro studies, however, are predominantly conducted using two-dimensional (2D) culture systems in which cells are grown on rigid planar substrates. While experimentally convenient, such systems lack physiologically relevant matrix architecture and mechanical context, potentially leading to altered cell morphology, signaling, and function. Indeed, discrepancies between macrophage behavior observed in vitro and in vivo have raised concerns that standard 2D culture conditions may incompletely recapitulate native macrophage biology [4].

To date, human monocytic THP-1 cells are a commonly used alternative to primary human monocytes for studying monocyte differentiation into macrophages and the resulting macrophage bioactivities. Although THP-1 cells are a suspension cell line originally isolated from the peripheral blood of a patient with acute monocytic leukemia and subsequently immortalized for experimental purposes, they remain widely used to investigate macrophage differentiation and functional responses to various biomaterials and drugs. Despite their extensive use in studies of macrophage differentiation [5], macrophage activity [6], and drug delivery [7], relatively little attention has been given to the differences between THP-1 cells cultured in 2D versus 3D environments.

Although it is well established that in vitro models incorporating 3D extracellular matrix (ECM) biomaterials can simulate aspects of native tissue architecture [8,9], it remains unclear how matrices with distinct biochemical compositions, structural organizations, and mechanical properties influence macrophage populations following THP-1 differentiation. Furthermore, the extent to which these matrix-dependent effects differ from macrophages cultured in conventional 2D systems remains poorly defined.

In addition to morphology and viability, macrophage phenotype is defined by expression of surface receptors, secretion of cytokines, and production of proteoglycans that regulate inflammatory mediator storage and release. Among these, serglycin and its glycosaminoglycan modifications play key roles in controlling cytokine bioavailability [10], yet little is known about how extracellular dimensionality influences its post-translational processing. Likewise, the extent to which spatial context affects macrophage–tumor cell interactions remains insufficiently characterized, despite the recognized importance of tumor-associated macrophages in cancer progression [11].

Here, we investigated how culture dimensionality and matrix composition regulate the phenotype and function of monocyte-derived macrophages. Using THP-1 cells differentiated into M0, M1, and M2 macrophages, we performed a systematic comparison of cells cultured under conventional 2D conditions or embedded within 3D matrices composed of either Matrigel, a basement membrane-like material, or type I collagen. We assessed morphological features, viability, membrane marker expression, cytokine production, proteoglycan modification, and antitumor activity in coculture with breast cancer cells. By integrating structural, molecular, and functional analyses, this study aims to define how extracellular architecture shapes macrophage biology and to determine whether 3D culture systems more faithfully reproduce physiologically relevant immune phenotypes than traditional planar platforms.

## 2. Materials and Methods

### 2.1. Cell Culture

Human monocytes (THP-1 cells) constitutively expressing GFP (Applied Biological Materials Inc., Richmond, BC, Canada, Cat. #T9616) were cultured in RPMI-1640 medium (Gibco, Waltham, MA, USA, Cat. #A1049101) supplemented with 10% fetal bovine serum (Gibco, Cat. #16000044) and 1% penicillin–streptomycin (Gibco, Cat. #15140122), and maintained at 37 °C in a humidified incubator with 5% CO_2_. This suspension cell line was cultured in untreated T25 flasks (Thermo, Waltham, MA, USA, Cat. #169900).

### 2.2. Macrophage Differentiation

The differentiation of THP-1 cells into M1 or M2 macrophages under 2D conditions was performed based on a previously published protocol [12]. Briefly, THP-1 cells were seeded at 1 × 10^6^ cells/mL in 96-well plates and differentiated into M0 macrophages by treatment with 150 nM phorbol 12-myristate 13-acetate (PMA; Sigma-Aldrich, St. Louis, MO, USA, Cat. #524400) for 24 h at 37 °C. Cells designated as M0 macrophages were maintained in medium containing PMA. Cells designated for M1 or M2 polarization were not subjected to medium replacement; instead, M0 macrophages were polarized to M1 macrophages by adding 20 ng/mL IFN-γ (PeproTech, Cranbury, NJ, USA, Cat. #300-02-20UG) and 10 pg/mL LPS (Sigma, Cat. #L7770) directly to the existing medium. M2 polarization was induced by adding 20 ng/mL interleukin-4 (PeproTech, Cat. #200-04-20UG) and 20 ng/mL interleukin-13 (PeproTech, Cat. #200-13-10UG). Medium was replaced every two days with fresh medium containing the same concentrations of PMA and polarization factors.

For 3D culture, 3 mg/mL Matrigel (Corning^®^, Corning, NY, USA, Cat. #354234) or type I collagen (ibidi, Fitchburg, WI, USA, Cat. #50201) was prepared. THP-1 cells (1 × 10^6^ cells/mL) were mixed with each matrix and seeded into 96-well plates. After 10–20 min of gelation, medium containing the same PMA concentration described above was added. After 24 h, samples designated for M1 or M2 differentiation were treated with the corresponding polarization factors described above. Both 2D and 3D cultures were monitored for 7 days and imaged using a fluorescence microscope (Nexcope, Ningbo, China, Cat. #NIB620-FL).

### 2.3. Cell Lysate Preparation

After 7 days of culture, cell lysates were collected for Western blot analysis according to a previously described protocol [13]. For 2D cultures, cells were washed with PBS, lysed in RIPA buffer (Merck, Darmstadt, Germany, Cat. #3519189), and scraped from the plate. Suspensions were transferred to microcentrifuge tubes and incubated on ice for 15 min. Samples were homogenized using an ultrasonic homogenizer (Branson, Danbury, CT, USA, Cat. #C9NB) for 20 s, followed by 1 min on ice; this cycle was repeated three times. Samples were then incubated on ice for an additional 15 min and centrifuged at 13,000× *g* for 5 min at 4 °C. Supernatants were collected and stored at −20 °C until analysis. For 3D cultures, cells were first released from matrices using collagenase digestion for 15 min at 37 °C, followed by centrifugation at 1000 rpm for 5 min. The resulting cell pellets were processed using the same lysis protocol described above.

### 2.4. Membrane Protein Purifications

THP-1-derived macrophage membrane proteins were separated from the rest of the cell components as described previously [14]. Briefly, cells in PBS were centrifuged at 1100× *g* for 15 min at room temperature, and then the pellet was resuspended in RIPA buffer. The solution was kept on ice for 30 min followed by sonication (6 × 15 s). The cell homogenate was centrifuged at 1500× *g* for 10 min, in which the separated cell organelles were discarded, and the rest of the protein component, in the supernatant, was kept. After centrifuging at 180,000× *g* for 2 h at 4 °C, the membrane proteins (in pellet form) were dissolved in Na_2_CO_3_ and centrifuged again at 180,000× *g* for 2 h at 4 °C.

### 2.5. SDS PAGE and Western Blotting

Gradient 4–12% SDS-polyacrylamide gels (Thermo, Cat. #HC2040) were prepared according to the manufacturer’s instructions using the Mini-PROTEAN^®^ Tetra Handcast System (Bio-Rad, Drive Hercules, CA, USA, Cat. #1658000FC). Samples were electrophoresed at 100 V at room temperature until the dye front reached the bottom of the gel. Proteins were transferred onto polyvinylidene fluoride (PVDF) membranes (Merck Millipore, Burlington, MA, USA, 0.45 µm; Cat. #IPVH00010).

After overnight blocking at 4 °C, membranes were incubated overnight at 4 °C with primary antibodies under gentle agitation. Membranes were washed with 0.1% TBST and incubated with secondary antibodies for 1 h at room temperature with shaking. Following additional washes, immunoreactive signals were detected using ECL Plus reagent (Merck Millipore, Cat. #WBKLS0500). Antibody information is provided in Supplementary Table S1.

### 2.6. ELISA

Human TNF-α, FGF2, and serglycin levels were quantified using commercially available ELISA kits. Supernatants collected from each group on Day 7 were analyzed using TNF-α (ABclonal, Woburn, MA, USA, Cat. #RK00030), FGF2 (Abcam, Waltham, MA, USA, Cat. #ab246531), and serglycin (ABclonal, Cat. #RK09141) kits according to the manufacturers’ protocols.

### 2.7. Macrophage–Cancer Cell Coculture

Human breast cancer cells (MDA-MB-231) constitutively expressing RFP (GenTarget, San Diego, CA, USA, Cat. #SC044) were cultured in DMEM (Gibco, Cat. #2705248) supplemented with 10% fetal bovine serum (Gibco, Cat. #16000044) and 1% penicillin–streptomycin (Gibco, Cat. #15140122). For 2D coculture, after THP-1 cells (1 × 10^5^ cells/mL) differentiated into M1 or M2 macrophages, MDA-MB-231 cells (1 × 10^5^ cells/mL) were added at a 1:1 ratio in a 50:50 mixture of DMEM and RPMI medium. For 3D coculture, macrophages were detached using Accutase (Merck, Cat. #SCR005), mixed with MDA-MB-231 cells in 3 mg/mL collagen, and seeded into 96-well plates. Cocultures were monitored and imaged using a fluorescence microscope (Nexcope, Cat. #NIB620-FL).

### 2.8. Statistical Analysis

Statistical significance among experimental groups was evaluated using one-way analysis of variance (ANOVA) followed by Tukey’s post hoc test. A *p* value < 0.05 was considered statistically significant.

## 3. Results

### 3.1. Dimensionality-Dependent Morphology and Viability in Matrigel Cultures

**Figure 1:**
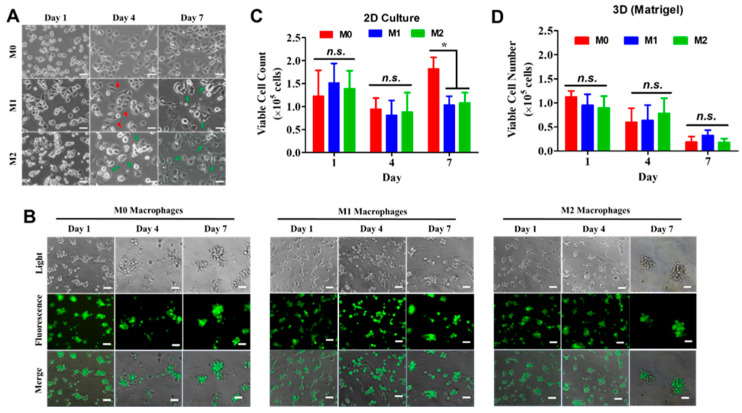
Morphological differences of monocyte-derived macrophages cultured in 2D and 3D Matrigel. (**A**) Human THP-1 monocytes were chemically induced to differentiate into M0, M1, or M2 macrophages on tissue culture plates. Spindle-like structures (red arrows) were observed in M1 macrophages on Day 4, whereas dendritic-like structures (green arrows) were observed on Day 7. Dendritic-like structures were observed in M2 macrophages on Days 4 and 7. (**B**) Representative images of M0, M1, and M2 macrophages cultured in 3 mg/mL Matrigel. Cells constitutively expressed GFP. Scale bar: 50 µm. (**C**,**D**) Quantification of viable cell numbers for M0, M1, and M2 macrophages cultured on tissue culture plates (2D) or in Matrigel (3D), N = 3. * *p* < 0.05; n.s., not significant.

The effect of culture dimensionality on monocyte-derived macrophages was first evaluated by morphological analysis. GFP-expressing human THP-1 monocytes were chemically induced to differentiate into M0, M1, or M2 macrophages either on tissue culture plates (2D) or in 3 mg/mL Matrigel (3D). A concentration of 3 mg/mL was selected as the working concentration for Matrigel because it is the minimum concentration required to form a stable gel in vitro [15]. In 2D culture, M0 macrophages transitioned from suspension to adherent cells and maintained a rounded morphology throughout 7 days. M1 macrophages developed spindle-like structures by Day 4 and dendritic-like morphologies by Day 7, whereas M2 macrophages displayed dendritic morphologies at both time points (Figure 1A).

In contrast, macrophages cultured in Matrigel exhibited predominantly rounded, aggregated morphologies regardless of phenotype throughout the culture period (Figure 1B). Fluorescence imaging indicated aggregate formation beginning on Day 4, accompanied by reduced apparent cell confluence over time. Quantification of viable cells in 2D culture showed that differentiated M1 and M2 macrophages had significantly lower viability than M0 macrophages (Figure 1C). Notably, macrophages cultured in Matrigel exhibited a progressive decline in viable cell numbers (>50%) over 7 days (Figure 1D). Consistent with imaging observations, total viable cell counts were substantially lower in 3D Matrigel cultures than in 2D conditions.

### 3.2. Collagen-Based 3D Culture Supports Macrophage Growth and Morphology

**Figure 2:**
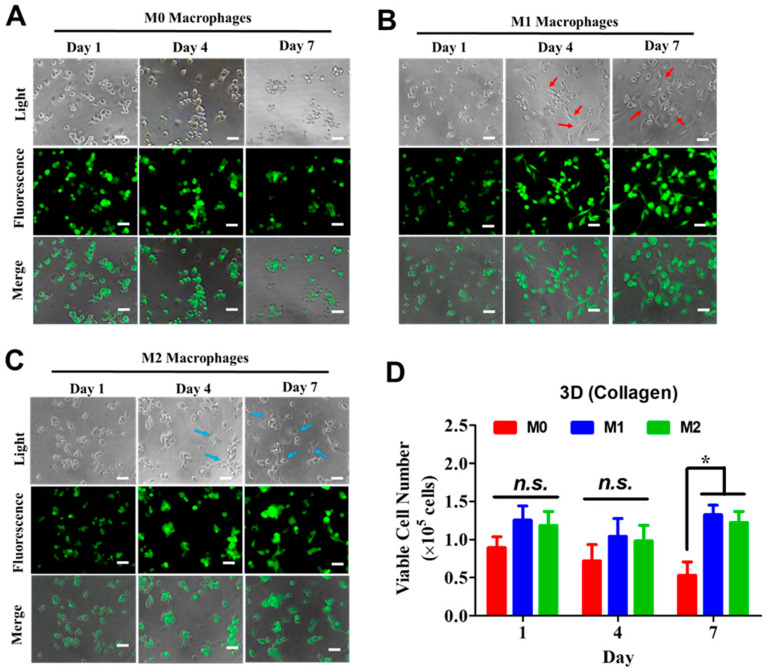
Morphological differences of monocyte-derived macrophages cultured in collagen gels. Human GFP-expressing THP-1 monocytes were cultured in collagen gels and chemically induced to differentiate into (**A**) M0 macrophages, (**B**) M1 macrophages, or (**C**) M2 macrophages. Spindle-like structures were observed in M1 macrophages (red arrows), whereas M2 macrophages predominantly exhibited dendritic-like morphologies (blue arrows). Scale bar, 50 µm. (**D**) Quantification and statistical analysis of viable cell numbers for each macrophage phenotype cultured in collagen gels (N = 3). * *p* < 0.05; n.s., not significant.

Since the Matrigel culture did not support sufficient cell growth under these conditions, subsequent 3D experiments were performed using collagen gels. Commercially available type I collagens were used to make 3 mg/mL collagen gels with macrophages embedded inside. M0 macrophages cultured in collagen retained rounded morphologies similar to those observed in Matrigel and formed aggregates over time, with reduced apparent confluence during the 7-day culture period (Figure 2A). In contrast, M1 macrophages displayed spindle-like morphologies from Day 4 onward (Figure 2B), while M2 macrophages developed dendritic-like structures beginning on Day 4 (Figure 2C). Both M1 and M2 macrophages exhibited markedly improved growth in collagen gels compared with Matrigel cultures. Quantification confirmed that M0 macrophage numbers were significantly lower than those of M1 or M2 macrophages in collagen gels (Figure 2D). Based on these results, all subsequent 3D experiments were performed using 3 mg/mL collagen gels.

### 3.3. Membrane Protein Expression Profiles Under 2D and 3D Conditions

**Figure 3:**
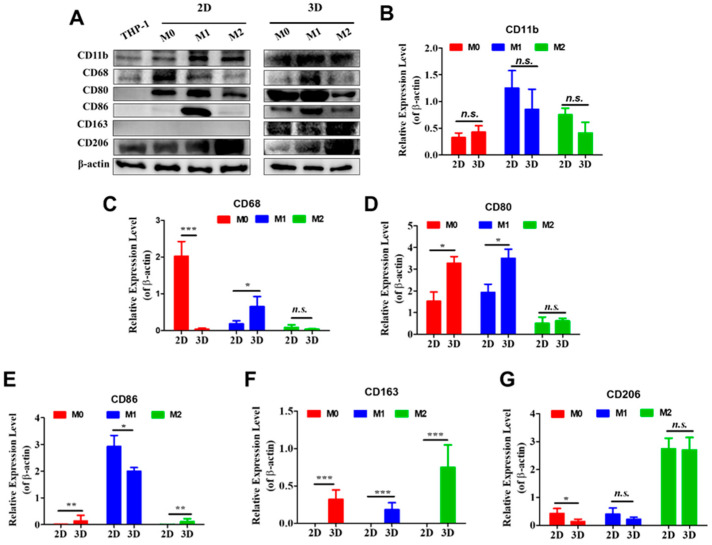
Expression of membrane proteins under 2D and 3D culture conditions. (**A**) Western blot analysis of membrane proteins expressed by different macrophage phenotypes cultured under 2D or 3D conditions for 7 days. (**B**–**G**) Statistical analysis of membrane protein expression levels. N = 3, * *p* < 0.05; ** *p* < 0.01; *** *p* < 0.001; n.s., not significant.

To determine whether culture dimensionality alters macrophage phenotype, membrane protein expression was analyzed. Cells cultured in 2D were harvested by scraping, and membrane proteins were isolated by ultracentrifugation before Western blot analysis (Figure 3A). For 3D cultures, cells were released from collagen gels using collagenase before membrane protein isolation. Undifferentiated THP-1 monocytes served as controls.

CD11b, a marker associated with pro-inflammatory macrophages [16], showed the highest expression in M1 macrophages, intermediate expression in M2 macrophages, and the lowest expression in M0 macrophages under both 2D and 3D conditions. No significant differences in CD11b expression were observed between dimensionalities for any subtype (Figure 3B).

CD68, a pan-macrophage scavenger receptor [17], displayed distinct patterns depending on culture format. In 2D cultures, M0 macrophages exhibited the highest CD68 expression, whereas M1 and M2 macrophages showed lower levels (Figure 3C). In contrast, in 3D collagen cultures, M1 macrophages displayed the strongest CD68 expression, while M0 and M2 macrophages showed relatively weak signals. Direct comparison revealed significantly higher CD68 expression in M0 macrophages cultured in 2D than in 3D, whereas M1 macrophages showed the opposite trend. No significant dimensionality-dependent difference was observed for M2 macrophages.

CD80 and CD86, costimulatory receptors associated with M1 polarization [18], exhibited subtype-specific expression patterns. CD80 was strongly expressed in M0 and M1 macrophages but weakly expressed in M2 macrophages under both culture conditions (Figure 3D). Dimensionality influenced CD80 expression in M0 and M1 macrophages, with significantly higher levels observed in 3D than in 2D cultures, whereas M2 macrophages showed no significant difference.

CD86 expression was highest in M1 macrophages in both culture formats (Figure 3E). In 2D culture, CD86 was detected primarily in M1 macrophages, whereas in 3D culture, it was detectable in all macrophage subtypes but remained strongest in M1 cells. Dimensionality comparisons showed significantly higher CD86 expression in M1 macrophages in 2D than in 3D, whereas M0 and M2 macrophages exhibited higher expression in 3D cultures.

Markers associated with M2 polarization and tumor-associated macrophages, CD163 and CD206 [19], also displayed dimensionality-dependent regulation. CD163 was undetectable in all macrophage subtypes cultured in 2D but was strongly expressed in all subtypes in 3D collagen cultures, with the highest levels observed in M2 macrophages (Figure 3F). CD206 was detected in all subtypes under both conditions and was consistently highest in M2 macrophages (Figure 3G). M0 macrophages expressed significantly higher CD206 levels in 2D than in 3D, whereas M1 and M2 macrophages showed no significant differences between dimensionalities.

### 3.4. Cytokine Expressions and Secretions

**Figure 4:**
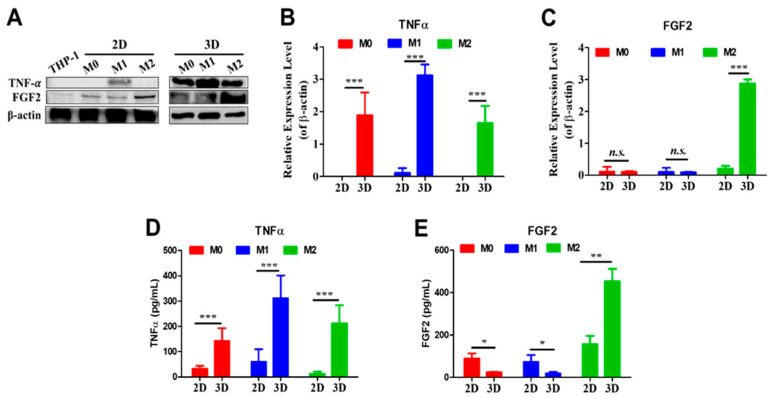
Expression of growth factors under 2D and 3D culture conditions. (**A**) Western blot analysis of growth factor expression in different macrophage phenotypes cultured under 2D or 3D conditions for 7 days. (**B**,**C**) Quantification of TNFα and FGF2 levels in cell lysates based on Western blot analysis. (**D**,**E**) Quantification of secreted TNFα and FGF2 levels in culture media collected on Day 7 under 2D or 3D conditions. N = 3, * *p* < 0.05; ** *p* < 0.01; *** *p* < 0.001; n.s., not significant.

To evaluate functional consequences of dimensionality, expression and secretion of TNF-α and FGF2 were analyzed (Figure 4A). TNF-α expression was highest in M1 macrophages in 2D cultures (Figure 4B). In cell lysates from 2D cultures, TNF-α was detectable only in M1 macrophages, whereas all macrophage subtypes cultured in collagen gels showed detectable TNF-α, with M1 macrophages exhibiting the highest levels. Overall, TNF-α expression in cell lysates was significantly higher in 3D cultures than in 2D cultures.

FGF2 expression was predominantly detected in M2 macrophages under both culture conditions, with minimal expression in M0 or M1 macrophages (Figure 4C). Expression levels of FGF2 in M2 macrophages were significantly higher in collagen cultures than in 2D cultures.

Analysis of secreted cytokines revealed that TNF-α was detectable in conditioned media from all macrophage subtypes under both culture conditions, even when intracellular TNF-α was undetectable (Figure 4D). In both 2D and 3D cultures, M1 macrophages secreted the highest levels of TNF-α. Regardless of subtype, TNF-α concentrations were significantly higher in media from 3D cultures than from 2D cultures.

In contrast, secreted FGF2 displayed subtype- and dimensionality-dependent patterns (Figure 4E). In 2D cultures, M0 and M1 macrophages secreted significantly more FGF2 than their counterparts in 3D cultures. However, M2 macrophages secreted significantly higher levels of FGF2 in collagen gels than in 2D conditions.

### 3.5. Serglycin Glycosylation Profiles Differ Between 2D and 3D Cultures

**Figure 5:**
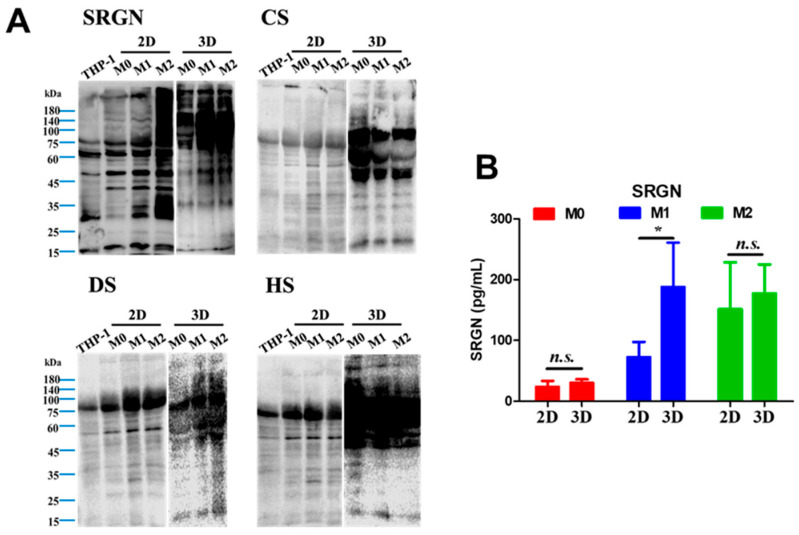
The protein expression patterns of serglycin and glycosaminoglycans in different macrophage populations under 2D and 3D culture conditions. (**A**) Western blot analysis of serglycin and three types of glycosaminoglycan expression in different macrophage phenotypes cultured under 2D or 3D conditions for 7 days. (**B**) Quantification of secreted serglycin levels in culture media collected on Day 7 under 2D or 3D conditions (N = 3). * *p* < 0.05; n.s., not significant.

Serglycin, a proteoglycan involved in cytokine storage and secretion [20], was analyzed to assess dimensionality-dependent changes in post-translational modification. In 2D cultures, M0 and M1 macrophages exhibited serglycin banding patterns similar to THP-1 monocytes, whereas M2 macrophages displayed smeared bands, indicating higher glycosylation levels (Figure 5A). Bands at ~15 kDa corresponded to the serglycin core protein.

In 3D cultures, serglycin from all macrophage subtypes migrated at higher molecular weights than in 2D cultures, indicating increased glycosylation. Consistent with this observation, core-protein signals were weaker in 3D samples, suggesting a greater proportion of highly modified serglycin species.

Analysis of glycosaminoglycan modifications revealed that chondroitin sulfate signals were stronger in macrophages cultured in collagen gels than in 2D cultures, and some bands did not overlap with serglycin signals, suggesting expression of additional proteoglycans. In contrast, dermatan sulfate signals were stronger in 2D cultures and appeared as discrete bands (~75 kDa and ~55 kDa), whereas 3D samples displayed broad smears. Similar patterns were observed for heparan sulfate.

ELISA analysis of conditioned media showed that M1 macrophages cultured in collagen gels secreted significantly more serglycin than those cultured in 2D (Figure 5B). M2 macrophages secreted high levels of serglycin under both conditions, with no significant difference between dimensionalities.

### 3.6. Dimensionality Influences Macrophage Antitumor Activity

**Figure 6:**
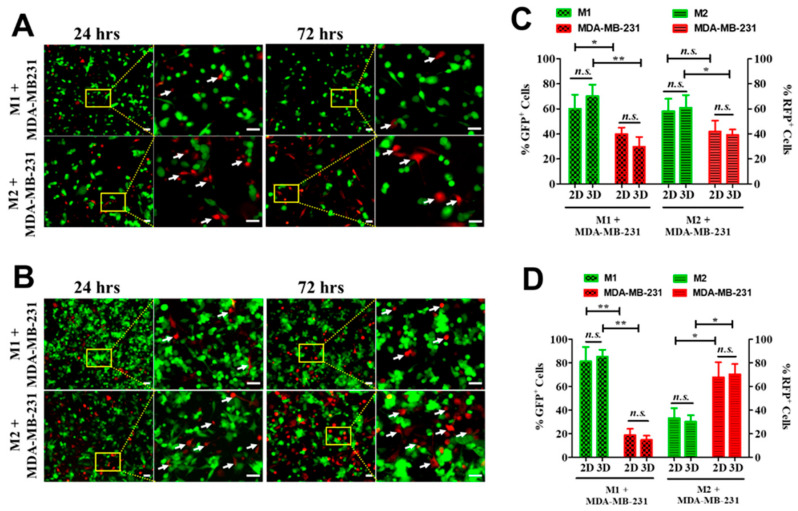
Coculture of monocyte-derived macrophages with human breast cancer cells. THP-1 cells were chemically induced to differentiate into M1 or M2 macrophages (green). Cells were then cocultured with human breast cancer cells, MDA-MB-231 (red, white arrows), for 24 or 72 h in either (**A**) tissue culture plates or (**B**) collagen gels. Scale bar, 50 µm. (**C**,**D**) Percentages of the two cell populations in coculture were analyzed at 24 h (**C**) and 72 h (**D**) by flow cytometry. 2D indicates coculture on tissue culture plates, and 3D indicates coculture in collagen gels (N = 3). * *p* < 0.05; ** *p* < 0.01; n.s., not significant.

To assess functional relevance, macrophage antitumor activity was evaluated by coculture with RFP-expressing human breast cancer cells (MDA-MB-231) for 24 h or 72 h. In 2D coculture, MDA-MB-231 cell numbers decreased over 72 h when cultured with M1 macrophages, whereas they increased when cocultured with M2 macrophages (Figure 6A). M1 macrophages adopted spindle morphologies, whereas M2 macrophages remained predominantly rounded.

In 3D collagen coculture, both macrophage subtypes formed large aggregates over 72 h (Figure 6B). Fluorescence imaging indicated fewer tumor cells in cocultures with M1 macrophages than with M2 macrophages at 72 h.

Flow cytometric quantification showed that at 24 h, M1 macrophages outnumbered tumor cells in both 2D and 3D cultures (Figure 6C). At the same time point, M2 macrophages exceeded tumor cell numbers only in 3D cultures. At 72 h, M1 macrophages remained significantly more abundant than tumor cells under both dimensionalities, whereas tumor cells significantly outnumbered M2 macrophages (Figure 6D).

## 4. Discussion

This study demonstrates that culture dimensionality and extracellular matrix composition are critical determinants of macrophage phenotype, functional polarization, and secretory behavior. By systematically comparing monocyte-derived macrophages cultured in conventional 2D systems with those maintained in physiologically relevant 3D matrices, we show that spatial context actively regulates membrane marker expression, cytokine production, proteoglycan modification, and antitumor activity. These findings establish that differentiation stimuli do not solely govern macrophage biology but are profoundly shaped by the structural properties of the surrounding microenvironment.

A principal observation is that 3D environments impose distinct morphological and viability constraints compared with 2D cultures. Matrigel cultures promoted aggregation and reduced viable cell numbers, whereas collagen matrices supported sustained growth and phenotype-specific morphologies. This was unexpected, as Matrigel is well known to promote cell growth and invasion in various cell types. Multiple factors likely contributed to this phenomenon. One possible explanation is that the composition of Matrigel is not macrophage-supportive. Matrigel is primarily composed of laminin, collagen IV, entactin, and growth factors, which mimic the basement membrane rather than interstitial tissue [21]. In vivo, macrophages typically reside and migrate within interstitial matrices rich in fibrillar type I collagen, not in basement membrane-like environments [22]. Moreover, the crosslinked basement membrane proteins presented in Matrigel could have resisted the degradation by macrophage-secreted proteases [23]. Hence, macrophages could not remodel the matrix; they would become physically constrained and metabolically suppressed. In contrast, collagen is a natural macrophage substrate and is readily remodeled by macrophage-secreted proteases such as MMPs and cathepsins. Additionally, macrophages rely on integrin-mediated adhesion signals (e.g., β1/β2 integrins) for survival and activation, and type I collagen is known to strongly support these interactions [24]. Thus, ECM ligands presented in Matrigel probably could not efficiently engage macrophage adhesion receptors, thereby reducing adhesion signaling that triggers anoikis-like responses or quiescence [25]. These findings suggested that matrix biochemical composition and mechanical properties differentially influenced macrophage survival and cytoskeletal organization. Since collagen is a major structural component of interstitial tissues, our results suggested that it provided adhesive ligands and mechanical cues that better recapitulated native macrophage niches, thereby enabling phenotypic features that were absent or attenuated in Matrigel or planar systems. The improved growth of M1 and M2 macrophages in collagen further indicated that matrix-specific signaling supports subtype-dependent adaptation.

Dimensionality-dependent regulation was also evident at the molecular level. Expression patterns of canonical surface markers demonstrated that some macrophage identifiers were stable across culture systems, whereas others were strongly microenvironment-sensitive. For example, CD11b expression followed expected subtype hierarchies regardless of dimensionality, indicating that core lineage identity was preserved. This receptor is predominantly expressed in M1 macrophages, with expression increasing as monocytes differentiate into mature M1 macrophages and decreasing upon polarization toward M2 macrophages [26], which matches the expression patterns observed in the Western blot analyses. In contrast, CD68, CD80, CD86, and CD163 exhibited marked shifts between 2D and 3D conditions, suggesting that activation state and functional readiness were modulated by spatial context. Interestingly, markers such as CD80 and CD206 are classical representatives of M1 and M2 macrophages, respectively; however, their expression was also detected in M0 macrophages under both 2D and 3D conditions. A recent study on the differentiation of peripheral monocytes in 2D culture similarly reported that M0 macrophages exhibited CD80 and CD206 expression levels comparable to those of M1 and M2 macrophages [27]. These observations support the concept that macrophage phenotypes exist along a continuum rather than as discrete states, and that marker expression is highly context-dependent.

Although CD68 is not a specific marker for M1 macrophages, its expression was upregulated in M1 macrophages compared with M2 macrophages [28]. This pattern was consistent with the Western blot results observed in 3D cultures of all three macrophage subtypes, but not in 2D cultures. Likewise, CD163 induction occurred exclusively in 3D culture and was absent under 2D conditions. This receptor is associated with tissue-resident and tumor-associated macrophages, implying that 3D matrices promote phenotypes that more closely resemble in vivo states [29]. Together, these data support a model in which macrophage identity reflects both intrinsic differentiation programs and extrinsic structural regulation.

Functional readouts further reinforced the importance of dimensionality. The proinflammatory cytokine TNF-α and the proangiogenic factor FGF2 showed subtype-specific expression patterns consistent with canonical M1 and M2 polarization, respectively. However, absolute expression levels and secretion profiles differed significantly between 2D and 3D systems. The observation that 3D cultures generally enhanced TNF-α production suggested that spatial confinement or matrix signaling amplified inflammatory activation pathways. Conversely, FGF2 secretion displayed context-dependent regulation, indicating that pro-reparative signaling was particularly sensitive to environmental architecture. Importantly, cytokine detection in conditioned media, even when intracellular levels were low, indicated that secretion dynamics could not be inferred solely from intracellular measurements, underscoring the necessity of functional assays when evaluating macrophage phenotypes.

A mechanistic insight emerging from this work was the dimensionality-dependent modification of serglycin, a proteoglycan that regulates the storage and release of inflammatory mediators and growth factors [30]. Macrophages cultured in 3D exhibited higher molecular weight serglycin species and broader glycosaminoglycan modification patterns, consistent with increased glycosylation complexity seen in vivo samples [31]. Since glycosaminoglycan chains control ligand binding and release kinetics [32], these modifications likely influenced cytokine retention and secretion efficiency. The higher molecular weight distribution of CS, DS, and HS in THP-1-derived macrophages cultured in 3D collagen suggests increased glycosylation, which may enhance cytokine-binding affinity and influence cellular bioactivity. The enhanced secretion of serglycin in 3D cultures, particularly in M1 and M2 macrophages, supports the concept that extracellular matrix architecture can regulate intracellular trafficking and secretory pathways [33]. Thus, structural context may modulate immune signaling not only at the transcriptional level but also through post-translational modification of key regulatory molecules.

The functional consequences of these phenotypic differences were evident in coculture experiments with breast cancer cells. M1 macrophages suppressed tumor cell abundance, whereas M2 macrophages supported tumor expansion, consistent with their established inflammatory versus reparative roles [34]. Notably, these effects were observed in both 2D and 3D systems but were more pronounced in 3D cultures, where macrophages formed aggregates and displayed spatial behaviors reminiscent of tissue infiltration [35]. Quantitative analysis confirmed that macrophage–tumor cell dynamics differed according to both polarization state and dimensionality, highlighting the importance of modeling immune–tumor interactions in structurally relevant environments. It would be of interest in the future to conduct additional assays, such as apoptosis or proliferation markers (e.g., cleaved caspase-3 or Ki-67), to further clarify whether dimensionality affects how THP-1-derived M1 macrophages restrict cancer cell growth.

Collectively, these findings provide evidence that 3D culture systems, particularly collagen-based matrices, more accurately reproduce physiologically relevant macrophage phenotypes than conventional 2D platforms. This has important implications for immunological research and drug discovery. Many screening pipelines rely on 2D macrophage assays [36], yet our data indicated that such systems could underestimate or misrepresent functional responses, especially those related to activation markers, cytokine secretion, and matrix-dependent signaling pathways. Incorporating 3D models could therefore improve predictive accuracy for therapeutic screening, reduce reliance on animal models, and facilitate mechanistic studies of immune regulation.

Several limitations should be considered. The study employed a macrophage system derived from a single monocyte cell line, and primary human macrophages may display additional heterogeneity [37]. Moreover, although collagen gels provide structural complexity, they do not fully replicate the biochemical diversity of native tissues. Some studies have demonstrated that collagen density does not affect macrophage proliferation or viability; however, it does influence gene expression [38,39]. Therefore, future work integrating multiple extracellular matrix components, tunable stiffness, and multicellular microenvironments will be valuable for dissecting how physical and biochemical cues cooperate to regulate macrophage behavior.

In summary, our results demonstrate that macrophage phenotype, signaling, and function are actively regulated by culture dimensionality and matrix composition. Three-dimensional collagen environments promote molecular and functional characteristics that more closely resemble physiological macrophage states, in part by modulating receptor expression, cytokine secretion, and serglycin glycosylation. These findings highlight spatial context as a central regulator of innate immune biology and support the use of structurally biomimetic culture systems as advanced platforms for mechanistic immunology studies and therapeutic development. Although THP-1-derived macrophages are a controlled and reproducible model, several limitations should be acknowledged. As an immortalized monocytic leukemia cell line, THP-1 cells do not fully recapitulate the heterogeneity, differentiation pathways, and functional complexity of primary human macrophages. Furthermore, PMA-induced differentiation may introduce baseline activation that does not reflect physiological conditions, and the resulting macrophages may exhibit altered gene expression, receptor profiles, and cytokine responses compared to primary cells. In addition, THP-1-derived macrophages lack donor variability and tissue-specific imprinting, which are important determinants of macrophage behavior in vivo. Consequently, while this model is well-suited for dissecting microenvironmental effects under controlled conditions, our findings should be interpreted with caution when extrapolating to primary human macrophages. Future studies incorporating primary cells and more complex tissue models will be essential to validate and extend these observations.

Another important limitation of this study is that key physicochemical properties of the Matrigel and collagen matrices, such as stiffness, porosity, and ligand composition, were not explicitly characterized. Macrophage behavior is highly responsive to both mechanical forces and biochemical cues. Therefore, we cannot rule out that the phenotypic differences observed between our culture conditions reflect matrix-specific biochemical and biophysical effects rather than dimensionality per se. As such, conclusions regarding the pure effect of 3D spatial geometry should be tempered. To fully decouple the influence of matrix architecture from structural mechanics and ligand presentation, future studies utilizing tunable engineered hydrogels are warranted.

## Data Availability

All materials are available from the corresponding author.

## Supplementary Materials

The following supporting information can be downloaded at: https://www.mdpi.com/article/10.3390/biomedicines14040882/s1, Table S1: List of antibodies.
