# Supplementary material for: Culture Dimensionality Regulates Protein Expression and Bioactivity in THP-1-Derived Macrophages
Source: Biomedicines. 2026 Apr 13;14(4):882. doi: 10.3390/biomedicines14040882 (PMC13114233; doi:10.3390/biomedicines14040882)
Supplement: Supplementary file 1 [file biomedicines-14-00882-s001.zip › biomedicines-4228335-supplementary.pdf]

Culture Dimensionality Regulates Protein Expression and Bioactivity in THP-1-Derived Macrophages

Shang-Wun Jhang[1], Liang-Fang Lin[2], Gizem Naz Canko[2,3], Bill Cheng[2]*

[1]Department of Neurosurgery, Changhua Christian Hospital, Taiwan

[2]Graduate Institute of Biomedical Engineering, National Chung-Hsing University, Taiwan

[3]Ph.D. Program in Tissue Engineering and Regenerative Medicine, National Chung-Hsing University, Taiwan

Correspondence
Bill Cheng, Ph.D.

Graduate Institute of Biomedical Engineering

National Chung-Hsing University

No.145, Xing Da Road, Taichung, 402, Taiwan

Phone: +886-4-22840165 #936

Email: bcheng@dragon.nchu.edu.tw

Keywords: Monocyte-derived macrophages, 2D & 3D cultures, protein expressions, bioactivity

Supplementary Material

Supplementary Tables

Supplementary Table S1. List of antibodies

Antibodies	Company	Catalogue Numbers	Dilution
CD11b	Arigobio	ARG22000	1:5000
CD68	Invitrogen	14-0688-82	1:5000
CD80	ABclonal	A16039	1:5000
CD86	CiteAb	A1199	1:5000
CD163	Invitrogen	14-1639-82	1:5000
CD206	ARP	E-AB-33717	1:5000
TNFα	Santa Cruz	sc-133192	1:2000
FGF2	Santa Cruz	sc-74412	1:2000
Serglycin	ABclonal	A6951	1:5000
Chondroitin sulfate	Merck Millipore	SAB4200696	1:2000
Dermatan sulfate	Merck Millipore	MABT819	1:2000
Heparan Sulfate	Merck Millipore	MAB2040	1:2000
B-actin	Genetex	GTX109639	1:10,000
